# Impact of Community-Based Medical Education Program Content on Medical Students’ Applications for Community Residency Positions

**DOI:** 10.7759/cureus.88174

**Published:** 2025-07-17

**Authors:** Tsuneaki Kenzaka, Shinsuke Yahata, Ayako Kumabe, Naoya Mizutani, Hogara Nishisaki, Masanobu Okayama

**Affiliations:** 1 Department of Internal Medicine, Hyogo Prefectural Tamba Medical Center, Tamba, JPN; 2 Division of Community Medicine and Career Development, Kobe University Graduate School of Medicine, Kobe, JPN; 3 Department of General Internal Medicine, Hyogo Prefectural Harima-Himeji General Medical Center, Himeji, JPN; 4 Division of Community Medicine and Medical Education, Kobe University Graduate School of Medicine, Kobe, JPN

**Keywords:** attitude, community-based medical education, community medicine, homestay, medium-term effect, residency program, rural area

## Abstract

Background: In this study, we compared four practice areas showing a medium-term effect of community-based medical education (CBME) with those that did not examine more effective practice content.

Methods: This observational study analyzed summer internship programs between 2015 to 2019 undertaken by Hyogo Prefecture community-based physicians who initiated training for regional quota students in 2017-2021. For applying to become a resident in each hospital, risk ratios (RRs) and 95% confidence intervals (CIs) were calculated based on the presence or absence of practice experience and grade of experience for each region. The content of practical CBME training in Tamba and three other regions was compared.

Results: Ninety-four participants were included (58 men and 36 women). CBME experience in Tamba during their senior years significantly increased the number of applicants for initial residency training compared with the number of participants who had no CBME experience in a determined region (RR=1.58, 95% CI: 1.04-2.39) and who experienced CBME during their junior years (RR=2.23, 95% CI: 1.15-4.33). In contrast to other regions, practice times in Tamba were longer for fostering empathy toward the community, understanding local healthcare needs, and building harmony with community residents.

Conclusions: CBME with such training content enhances interaction with residents, thus demonstrating its educational value for the first time. As a next step, we should consider interviewing participants directly about their interactions with residents and whether CBME influenced their choice of place of employment.

## Introduction

Community-based medical education (CBME) is a globally practiced approach in which medical students receive training within their local communities [1-3]. Through CBME, medical students develop various skills and ethical competencies in areas such as primary care [1,2] and rural healthcare [4,5].

CBME has been conducted and extensively implemented in Japan [6]. The Japanese Model Core Curriculum for Medical Education, developed in 2001, first incorporated CBME in its 2007 revision [7]. Subsequently, various Japanese studies have examined its educational impact and confirmed only short-term effects after practical training, with no evidence of medium- to long-term effects [8-11]. However, Kenzaka et al. [12] found medium-term educational effects of CBME [12]; the authors observed that medical students who participated in a short CBME program (three days, two nights) in Tamba City, Hyogo Prefecture, and at the Hyogo Prefectural Tamba Medical Center were significantly more likely to apply for initial training at this hospital, the primary medical hospital of the region [12]. The primary objective of conventional CBME is to visit a rural area, where the students toured hospital facilities, listened to senior doctors’ experiences, and practiced hands-on medical care, without close involvement with local patients and residents. This practice in Tamba included a two-day and one-night homestay at a local resident’s home. Kenzaka et al. surmised that this will contribute to providing opportunities for long-term contact with local residents and may contribute to the training of healthcare professionals who provide primary care and community medicine in rural areas [12]. However, this observation requires verification via comparative studies.

In this study, we assessed whether a medium-term educational effect was observed across the four regions, including Tamba, where significant differences in residency have been found [12]. Additionally, we clarified characteristics of the CBME content in Tamba. Specifically, we examined the specific program content in the Tamba region, which exhibited mid-term educational effects of CBME, as well as the specific program content in other practice regions, which did not exhibit any such effects. This was done to verify the effective training content and confirm the mid-term educational effects of CBME.

## Materials and methods

Study design and participants

This cohort study included regional quota students from Hyogo Prefecture who graduated from medical school and began their initial training between 2017 and 2021. The study was approved by the Ethics Committee of Hyogo Prefectural Tamba Medical Center, Tamba, Japan (approval number: Tan-I number 1061). The study was conducted in accordance with the Declaration of Helsinki (as revised in 2013). All the participants provided written informed consent to participate in the study.

Regional quota students and community medicine summer seminar

Participants were regional quota students who were expected to work in the Hyogo Prefecture. These students received a six-year medical scholarship from the Hyogo Prefecture, which was waived if they practiced in rural areas of the prefecture for nine years after becoming a doctor [12]. Approximately 70%-80% of the regional quota of students enrolled each year attended the community medicine summer seminar as part of their practical training. Participation in CBME was voluntary and had no impact on grades or academic progression [12].

The objectives of this CBME were as follows: (1) to understand the local role of each medical institution through experience in community medical activities provided by medical institutions in rural areas; (2) to deepen exchanges with regional quota students; (3) to deepen exchanges between regional quota students, quota doctors, and the local medical personnel; and (4) to learn about the people, culture, and customs of the area where the trainees may work in the future.

The training content was created by the host institution in line with the above objectives. The faculty would judge whether the content of the training was in line with the above objectives and may request revisions. However, faculty were not directly involved in the details of the training content itself and did not engage in outreach activities. During the annual orientation, the purpose of the CBME was fully explained to participating students.

Each year, a regional quota of students received CBME in one of the seven regional areas of Hyogo Prefecture. The areas to receive CBME were randomly allocated. Once a student participated in a rural area, they were not allowed to go to the same area again in the following year as part of the CBME exercise. The random allocation method was chosen by the organizers' administrative staff, who were not involved in the internship itself or its contents, considering the number of people in the host region. In this case, the internship site was decided without any regard to the university or cohort in which the intern was enrolled. This is because CBME provides an opportunity for regional quota students to learn about areas where they may have future work opportunities and to learn about different areas.

Methods

Participants were offered a CBME summer seminar in Hyogo Prefecture during their medical school years. The seminar was held simultaneously at seven locations in the prefecture every summer. Practice sites were selected randomly, and students could not visit the same site more than once. Most CBME programs included off-the-job training, health lectures, social events with meals shared with residents, and visits to important local historical sites.

The regions considered for the study were Tamba (Hyogo Prefectural Tamba Medical Center, 320 beds), Nishiwaki (Nishiwaki Municipal Hospital, 320 beds), Shiso (Shiso Municipal Hospital, 199 beds), and Ako (Ako City Hospital, 360 beds).

Participant characteristics included sex, university attended, whether they had practical training experience in each of the four regional hospitals (Hyogo Prefectural Tamba Medical Center, Nishiwaki Municipal Hospital, Shiso Municipal Hospital, and Ako City Hospital), and the year of studies during which the training occurred. Additionally, their aspirations to become general practitioners were assessed.

Exposure factors included the following: 1) experience of CBME versus no experience of CBME in the relevant region; 2) experience in senior years (four to six years of medical school) versus no experience of CBME in the relevant region and experience in junior years (one to three years of medical school); and 3) experience in senior years versus experience in junior years.

Participants were divided on the basis of the presence or absence of experience with CBME into a group that had experienced CBME in the relevant region and a group that had not experienced CBME in the relevant region (this did not mean that there was no experience with CBME itself, as all regional quota medical students had experienced at least one CBME in six years).

**Figure 1:**
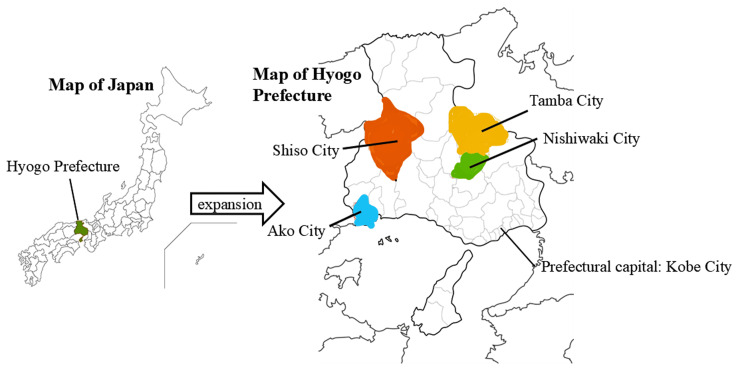
Four regions of the Hyogo Prefecture Tamba City, located in the mountains, has a population of approximately 60,000 and an area of 493.21 km². Nishiwaki City, located in the mountains, has a population of approximately 38,000 and an area of 132.44 km². Shiso City, located in the mountains, has a population of approximately 31,000 and an area of 658.54 km². Ako City, located on the seaside, has a population of approximately 43,000 and an area of 126.85 km² [13,14]. Blank map downloaded from https://www.chizu-seisaku.com/shirochizu/shirochizu-j-hyogo/. The authors have added the labels and highlighted the regions on the map.

Table 1 illustrates the distribution of regional quota medical students’ participation in CBME across the academic years. Figure 1 shows four regions in Hyogo Prefecture (Tamba, Nishiwaki, Shiso, and Ako). Table 2 presents the CBME implementation schedule and the process of selecting initial training hospitals.

**Table 1:** Diagram of CBME locations for regional quota medical students by academic year All 94 regional quota medical students have experienced at least one CBME in six years. CBME programs are offered annually to students in seven rural areas in Hyogo Prefecture, Japan. Areas are allocated irrespective of the students’ wishes, and no area is visited more than once. CBME: community-based medical education

Student number	Student 1	Student 2	Student 3	Student 4
First year	Tamba	Area D	Area F	Abstention
Second year	Nishiwaki	Nishiwaki	Ako	Shiso
Third year	Abstention	Area E	Area E	Ako
Fourth year	Shiso	Tamba	Shiso	Abstention
Fifth year	Ako	Area F	Area D	Area E
Sixth year	Abstention	Shiso	Abstention	Abstention

**Table 2:** Schedule for CBME implementation and selection of the initial training hospital The school year begins in April and ends in March. The placement of CBME in CMSS will be randomly selected from one of seven rural areas. Regional quota students may work at any of the 10 initial training hospitals in Hyogo Prefecture as doctors. They indicate their preferred hospital in April when they are in their sixth year of medical school. Hyogo Prefecture decides where they will work for their initial training based on the wishes of the regional quota students in July. CBME: community-based medical education; CMSS: community medicine summer seminar

Month	April	May	June	July	August	September –March
First to sixth year of medical school	－	Registration for CBME in CMSS	－	Placement will be randomly selected from one of seven regions	Experience of CBME in CMSS for 3 days/2 nights	－
Sixth year of medical school	Apply for an initial training hospital as a resident doctor	－	－	Determination of the initial training hospital as a resident doctor	－	－
First-year resident doctor	Become a resident doctor at an assigned initial training hospital	－	－	－	－	－

Whether the students applied for initial training at the respective hospitals and started their first residency at the respective hospitals was the analyzed outcome.

Regional quota students can work at any of the 10 training hospitals in Hyogo Prefecture. In their sixth year of studies, they submit their work placement preferences for approximately three of these hospitals. Hyogo Prefecture then assigns the hospital for initial training based on the students’ preferences.

The program content of the 2015-2019 CBME for regional quota students was examined. Three factors (I to III) have been determined to motivate medical students toward community medicine, which can be further categorized into nine subcategories ((1) to (9)) [15]: I. “envisioning and preparing for practicing community healthcare,” defined as (1) “empathy for the community,” (2) “understanding the demands for community healthcare,” (3) “understanding the practices of community healthcare,” (4) “finding a role model,” and (5) “diminishing conflicts between personal life and career;” II. “Belonging to a supportive community” was subdivided into (6) “robust construction of students’ community healthcare” and (7) “harmonization with community residents”; III. “Psychological effects” were subdivided into (8) “affect heuristic” and (9) “framing effect.”

The following contents are suggested to be included in the CBME program: increase opportunities to interact with residents (items 1, 2, 7, and 8); have residents share their appreciation of medical personnel (items 2 and 7); have local medical personnel share the rewards and attractions of their work and personal lives (items 1, 5, and 9); provide time for casual and enjoyable exchanges among students (items 6 and 8); and allow time to experience the local environment and culture in an enjoyable way (items 1 and 8) [15].

In our studies’ classification of CBME programs, the program content was divided into seven categories, excluding items (8) and (9). Items (8) “affect heuristic” and (9) “framing effect” were excluded because they stem from personal feelings and are difficult to classify based on the training content. Time spent sightseeing was categorized as (1) and (6). For instances where students were expected to interact with each other during their stay at the hotel, the exact time until they interacted was unknown. For convenience, the time was determined to be until 23:00 and categorized as (6). The program contents were evaluated by two researchers specializing in medical education to determine which items were applicable. These researchers were independent of each CBME program, including that in Tamba. Some program contents applied to over one item, and in cases where the evaluation differed, the decision was made through discussion. Seven content items were offered at the four hospitals between 2015 and 2019. The time (minutes) for each experiment was calculated and tabulated. The schedule for practical training and hands-on experience at Tamba in 2019 is presented in Appendix A.

An example of the classification of CBME programs based on the specific training content in the Tamba schedule in 2019 showed the following.

Empathy for the Community

The mayor talks about the city’s attractions and medical care issues; Gathering with regional quota doctors, hospital staff, and city hall staff in the area (including food and drink); Homestay at a local resident’s home (including meals, bathing, and health interviews with students); Students interview local staff who are from Tamba; Visit to famous places in the area; Students participate in local community activities, taking blood pressure measurements, interviewing residents with health-related questions, conducting exercises, hosting tea parties, and giving talks to residents; Under the guidance of local instructors, students participate in farming activities (e.g., planting vegetables) and fish for “amago,” a type of red-spotted trout, and gather (including meals and drinks) with hospital doctors, local medical personnel (including doctors, nurses, pharmacists, and office staff), and residents.

Grasping the Demands for Community Healthcare

The mayor gives a lecture on the city’s attractions and medical care issues; Gathering with regional quota doctors, hospital staff, and city hall staff in the area (including food and drink); Homestay at a local resident’s home (including meals, bathing, and health interviews with students); Students interview local staff who are from Tamba; Group discussion and presentation by students and hospital staff regarding medical issues in the area and their solutions; Students participate in local community activities, taking blood pressure measurements, interviewing residents with health-related questions, conducting exercises, hosting tea parties, and giving talks to residents; and gathering (including meals and drinks) with hospital doctors, local medical personnel (including doctors, nurses, pharmacists, and office staff), and residents.

Understanding the Practices of Community Healthcare

Medical experience training such as cardiopulmonary resuscitation, simulator training, and mock interviews; Round table conference with regional quota doctors; Lecture by the president of Tamba Medical Center concerning the hospital’s overview and appeal; Students interview local staff who are from Tamba; Group discussion and presentation by students and hospital staff on the medical issues in the area and their solutions, and students participate in local community activities, taking blood pressure measurements, conducting health interviews and exercises, hosting tea parties, and giving talks to residents.

Finding a Role Model

This included a round table conference with regional quota doctors.

Diminishing the Conflicts Between Personal Life and Career

A round table conference with regional quota doctors was held.

Robust Construction of Students’ Community Healthcare

Group discussions and presentations by students and hospital staff on the area’s medical issues and their solutions; Under the guidance of local instructors, students experienced farming (e.g., planting vegetables) and catching amago (fish); Gathering (including meals and drinks) with hospital doctors, local medical personnel (including doctors, nurses, pharmacists, and office staff), and residents; Interaction and exchange of opinions between students in the regional quota at the local inn where they will be staying; and a tour of the area’s famous sites.

Harmonization with Community Residents

Gathering with regional quota doctors, hospital staff, and city hall staff in the area (including food and drink); Homestay at a local resident’s home (including meals, bathing, and students' interviews with residents about their health); Students interview local staff who are from Tamba; Group discussions and presentations by students and hospital staff about the medical issues in the area and their solutions; Students participate in local community activities, taking blood pressure measurements, interviewing residents concerning their health, conducting exercises, hosting tea parties, and giving talks to residents; Under the guidance of local instructors, students participate in farming (e.g., planting vegetables) and fish for amago, and gather (including meals and drinks) with hospital doctors, local medical personnel (including doctors, nurses, pharmacists, and office staff), and residents.

Data analysis

Participant characteristics were tabulated for each of the four regions. We calculated risk ratios (RRs) and 95% confidence intervals (CIs) for each exposure factor using Poisson regression analysis with robust standard errors [16] against the outcome measures, adjusting for sex, university of origin, and whether the participant expressed an interest in becoming a general practitioner at the time of matching as confounding factors.

RRs with 95% CIs not including one were considered statistically significant. The outcome measures were as follows: 1) applying to become a resident in each hospital and 2) becoming a resident in each hospital.

The time for each program content item (1)-(7) was then tabulated. The average time per CBME practical training session between 2015 and 2019 was calculated, and the practical training time in the Tamba region was compared with that in other regions using the Mann-Whitney U test (accounting for overlapping time within a single training session). Differences were considered statistically significant at p < 0.05. All statistical analyses were performed using Stata MP, version 15 (StataCorp, College Station, TX, USA).

## Results

Table 3 presents the demographics of the students who participated in CBME across the four hospital areas. Overall, 94 participants were included: 58 (61.7%) were men, and 36 (38.3%) were women.

**Table 3:** Basic participant demographics * Data from four participants were missing.

Area	Tamba		Nishiwaki		Shiso		Ako	
Experience or Control	Experience	Control	Experience	Control	Experience	Control	Experience	Control
n	n = 37	n = 57	n = 24	n = 70	n = 30	n = 64	n = 11	n = 83
ー	n (%)	n (%)	n (%)	n (%)	n (%)	n (%)	n (%)	n (%)
Sex (male)	22 (59.5)	36 (63.2)	13 (54.2)	45 (64.3)	16 (53.3)	42 (65.6)	6 (54.6)	52 (62.7)
University	ー	ー	ー	ー	ー	ー	ー	ー
Jichi Medical University	6 (16.2)	6 (10.5)	1 (4.2)	11 (15.7)	5 (16.7)	7 (10.9)	1 (9.1)	11 (13.3)
Hyogo College of Medicine	9 (24.3)	12 (21.1)	2 (8.3)	19 (27.1)	3 (10.0)	18 (28.1)	1 (9.1)	20 (24.1)
Kobe University	20 (54.1)	25 (43.9)	18 (75.0)	27 (38.6)	17 (56.7)	28 (43.8)	7 (63.6)	38 (45.8)
Tottori University	1 (2.7)	9 (15.8)	2 (8.3)	8 (11.4)	4 (13.3)	6 (9.4)	1 (9.1)	9 (10.8)
Okayama University	1 (2.7)	5 (8.8)	1 (4.2)	5 (7.1)	1 (3.3)	5 (7.8)	1 (9.1)	5 (6.0)
General practice intention*	12 (33.3)	19 (35.2)	9 (37.5)	22 (31.4)	11 (36.7)	20 (31.2)	3 (27.3)	28 (33.7)
Applying to become a resident doctor in each hospital	18 (48.7)	27 (47.4)	15 (62.5)	36 (51.4)	2 (6.7)	2 (3.1)	9 (81.8)	41 (49.4)
Becoming a resident doctor in each hospital	8 (21.6)	9 (15.8)	6 (25.0)	14 (20.0)	3 (10.0)	5 (7.8)	1 (9.1)	14 (16.9)

Table 4 presents the RRs for students with CBME experience versus those without, across the four hospital regions. No significant differences were found in the RRs for either applying to become a resident or for becoming a resident.

**Table 4:** Influence of an undergraduate CBME program in each of the four hospital areas: RRs for students with CBME experience in Tamba compared to those without CBME: community-based medical education; CI: confidence interval; RR: risk ratio * Adjusted for sex, university, and general practice intentions in the final year of studies.

Aera	Tamba		Nishiwaki		Shiso		Ako	
95％ CI	RR (95% CI)	Adjusted RR* (95% CI)	RR (95% CI)	Adjusted RR* (95% CI)	RR (95% CI)	Adjusted RR* (95% CI)	RR (95% CI)	Adjusted RR* (95% CI)
Applying to become a resident doctor in each hospital	1.03 (0.67 to 1.58)	1.17 (0.74 to 1.84)	1.22 (0.83 to 1.79)	1.29 (0.86 to 1.93)	2.13 (0.32 to 14.43)	3.31 (0.55 to 20.01)	1.66 (1.16 to 2.36)	1.82 (1.27 to 2.62)
Becoming a resident doctor in each hospital	1.37 (0.58 to 3.23)	1.26 (0.54 to 2.97)	1.25 (0.54 to 2.89)	1.07 (0.48 to 2.39)	1.28 (0.33 to 5.01)	1.99 (0.53 to 7.43)	0.54 (0.08 to 3.71)	0.57 (0.05 to 6.39)

Table 5 presents the RRs comparing students who experienced CBME during their senior years (four to six years of medical school) with those who experienced CBME during their junior years (one to three years of medical school) and had no CBME experience in a determined region. The adjusted risk ratio (ARR) was 1.58 (95% CI: 1.04-2.39), indicating that students who experienced CBME during their fourth to sixth year of studies were significantly more likely to enter a residency program in Tamba. However, no significant differences were found among the three hospitals outside Tamba.

**Table 5:** Influence of experience in senior years (four to six years of medical school) versus no experience of CBME in the relevant region and experience in junior years (one to three years of medical school): RRs for participants with CBME experience in each of the four regional hospitals during their senior years (four to six years of medical school) compared with those of participants who experienced CBME during their junior years (one to three years of medical school) and had no CBME experience in a determined region CBME: community-based medical education; CI: confidence interval; RR: risk ratio * Adjusted for sex, university, and general practice intentions in the final year of studies. In Ako, the number of cases of “Becoming a resident” (N = 1) was significantly low for statistical analysis.

Area	Tamba		Nishiwaki		Shiso		Ako	
95％ CI	RR (95% CI)	Adjusted RR* (95% CI)	RR (95% CI)	Adjusted RR* (95% CI)	RR (95% CI)	Adjusted RR* (95% CI)	RR (95% CI)	Adjusted RR* (95% CI)
Applying to become a resident doctor in each hospital	1.68 (1.13 to 2.50)	1.58 (1.04 to 2.39)	1.13 (0.71 to 1.79)	1.28 (0.81 to 2.02)	1.32 (0.14 to 11.95)	1.59 (0.21 to 12.14)	1.28 (0.70 to 2.32)	1.36 (0.76 to 2.45)
Becoming a resident doctor in each hospital	1.60 (0.60 to 4.24)	1.78 (0.69 to 4.59)	0.59 (0.15 to 2.26)	0.62 (0.19 to 2.04)	1.32 (0.29 to 6.01)	1.39 (0.38 to 5.09)	－	－

Table 6 presents the RRs comparing students who experienced CBME during their senior years (four to six years of medical school) with those who experienced CBME during their junior years (one to three years of medical school) in each of the four regional hospitals. The ARR was 2.23 (95% CI: 1.15-4.33), indicating that students who experienced CBME in senior years (four to six years of medical school) typically entered a residency program in Tamba. However, no significant differences were found among the three hospitals outside Tamba.

**Table 6:** Influence of experience in senior years (four to six years of medical school) versus experience in junior years (one to three years of medical school): RRs for participants who experienced CBME in each of the four regional hospitals during their senior years (four to six years of medical school) compared with those of participants who experienced CBME in their junior years (one to three years of medical school) CBME: community-based medical education; CI: confidence interval; RR: risk ratio * Adjusted for sex, university, and general practice intentions in the final year of medical studies. The Adjusted RRs of Shiso and Ako were not statistically calculated because of small sample sizes. In Ako, the number of cases of “Becoming a resident” (N = 1) was significantly low for statistical analysis.

Area	Tamba		Nishiwaki		Shiso		Ako	
95% CI	RR (95% CI)	Adjusted RR* (95% CI)	RR (95% CI)	Adjusted RR* (95% CI)	RR (95% CI)	Adjusted RR* (95% CI)	RR (95% CI)	Adjusted RR* (95% CI)
Applying to become a resident doctor in each hospital	2.30 (1.16 to 4.57)	2.23 (1.15 to 4.33)	0.97 (0.52 to 1.80)	0.95 (0.48 to 1.87)	0.58 (0.04 to 8.36)	－	0.67 (0.38 to 1.17)	－
Becoming a resident doctor in each hospital	1.47 (0.43 to 4.97)	3.13 (0.55 to 17.90)	0.42 (0.09 to 1.89)	0.54 (0.16 to 1.77)	1.16 (0.12 to 11.35)	－	－	－

The content of each practice conducted at each of the four hospitals from 2015 to 2019 was divided into seven categories. Table 7 presents the calculated hours (in minutes) for each of the seven content categories at the four hospitals, organized by year.

**Table 7:** Time for each of the seven content items provided at the four hospitals between 2015 and 2019, organized by year (1) “Empathy for the community,” (2) “grasping the demands for community healthcare,” (3) “understanding the practices of community healthcare,” (4) “finding a role model,” (5) “diminishing the conflicts between personal life and career,” (6) “robust construction of students’ community healthcare,” and (7) “harmonization with community residents.”

Year	2015				2016			
Area	Tamba	Nishiwaki	Shiso	Ako	Tamba	Nishiwaki	Shiso	Ako
Total number of days of practical training (days)	2	1	1	1	2	2	3	3
Content (1) (min)	300	0	0	0	300	340	400	650
Content (2) (min)	180	0	0	0	180	220	240	660
Content (3) (min)	435	135	230	120	240	170	610	320
Content (4) (min)	75	30	0	0	60	0	70	0
Content (5) (min)	75	30	0	0	60	0	50	0
Content (6) (min)	120	0	0	0	340	120	360	130
Content (7) (min)	155	0	0	0	275	295	360	520
Total	1,340	195	230	120	1,455	1,145	2,090	2,280
Year	2017				2018			
Area	Tamba	Nishiwaki	Shiso	Ako	Tamba	Nishiwaki	Shiso	Ako
Total number of days of practical training (days)	3	3	3	0	3	3	3	3
Content (1) (min)	895	300	495	-	835	330	565	250
Content (2) (min)	730	270	270	-	745	240	310	210
Content (3) (min)	455	630	625	-	450	480	685	555
Content (4) (min)	30	60	70	-	30	180	50	45
Content (5) (min)	30	60	70	-	30	180	50	45
Content (6) (min)	615	510	745	-	580	590	785	490
Content (7) (min)	790	270	240	-	825	210	280	210
Total	3,545	2,100	2,515	-	3,495	2,210	2,725	1,805

Year	2019			
Area	Tamba	Nishiwaki	Shiso	Ako
Total number of days of practical training (days)	3	3	3	3
Content (1) (min)	835	330	565	250
Content (2) (min)	745	240	310	210
Content (3) (min)	450	480	685	555
Content (4) (min)	30	180	50	45
Content (5) (min)	30	180	50	45
Content (6) (min)	580	590	785	490
Content (7) (min)	825	210	280	210
Total	3,495	2,210	2,725	1,805

Table 8 presents the results of the Mann-Whitney U test of practice time in the Tamba region and other regions by calculating the mean of the time (in minutes) for each of the seven content items provided at the four hospitals between 2015 and 2019. Owing to the small number of cases, the only statistically significant difference between Tamba and Ako was (1) “empathy for the community,” which was compared between the two groups. However, in the Tamba region, practice time for (1) “empathy for the community,” (2) “understanding community healthcare demands,” and (7) “harmonization with community residents” was longer than that in other regions.

**Table 8:** Practice time in Tamba and other regions for each of the seven content items provided at the four hospitals between 2015 and 2019 (1) “Empathy for the community,” (2) “grasping the demands for community healthcare,” (3) “understanding the practices of community healthcare,” (4) “finding a role model,” (5) “diminishing the conflicts between personal life and career,” (6) “robust construction of students’ community healthcare,” and (7) “harmonization with community residents.” The gray boxes indicate the areas where the time spent in each practice session was the longest.

Area	Tamba	Nishiwaki		Shiso		Ako	
Practical time (min)	Practical time (min)	Practical time (min)	vs. Tamba p-value	Practical time (min)	vs. Tamba p-value	Practical time (min)	vs. Tamba p-value
Content (1) (min)	642	257	0.245	387	0.346	287	0.048
Content (2) (min)	513	191	0.344	226	0.343	285	0.323
Content (3) (min)	407	379	0.599	559	0.116	395	0.806
Content (4) (min)	45	90	0.588	52	0.592	26	0.379
Content (5) (min)	45	90	0.588	48	0.750	26	0.379
Content (6) (min)	451	362	0.401	513	0.347	272	0.220
Content (7) (min)	567	197	0.115	232	0.248	205	0.085

## Discussion

This study revealed two new findings. First, the medium-term effect on the workplace choice was found only at Hyogo Prefectural Tamba Medical Center. In RRs and 95% CIs, which were adjusted for confounding factors such as sex, home university, and whether the participant expressed interest in becoming a general practitioner when matched by the student, applying to become a resident doctor in each hospital was significantly higher only in Tamba (Tables 5, 6). Therefore, only students who practiced CBME in Tamba in their senior year (four to six years of medical school) significantly applied to become resident doctors in Tamba. Participants who experienced CBME in their fourth to sixth year of medical studies were significantly more likely to be matched for initial training at this center, an effect not observed in other hospitals of similar size. Because the place of employment is ultimately decided by the Hyogo Prefecture, the final placement does not fully reflect the students' wishes. This is why, although there was a significant difference in "Applying to become a resident doctor in each hospital" in Tamba, there was no significant difference in "Becoming a resident doctor in each hospital." Second, the factors (1) “empathy for the region,” (2) “understanding the demands for community healthcare,” and (7) “harmonization with community residents” were significantly longer in the hospital of the Tamba region than in the other three regional hospitals.

CBME, which is generally conducted in Japan for one to two weeks, showed short-term effects after practical training. However, no medium- to long-term effects were observed [8-10]. In the current study, we conducted CBME for a short period of three days and two nights. Except for the Tamba region, such short-term practical training did not elicit the medium-term educational effects of CBME, as shown in previous studies in Japan [12]. The Rural Physician Associate Program [17], Parallel Rural Community Curriculum [18], and University of Missouri Rural Track Pipeline Program [19] have demonstrated the medium- to long-term educational impacts of CBME. These programs provide CBME in residences for extended periods (six to 54 weeks) with adequate financial support. To show the medium-term effects over several years of CBME in such a short duration of three days and two nights, it is insufficient to simply offer CBME to medical students, but rather the specifics of their CBME program.

Previous research [16] has shown that the factors that motivate medical students toward community medicine include “empathy for the community,” “understanding the demands for community healthcare,” “understanding the practices of community healthcare,” “finding a role model,” “diminishing the conflicts between personal life and career,” “robust construction of students’ community healthcare,” “harmonization with community residents,” “affect heuristic,” and “framing effect.” The present study revealed that among these factors, “empathy for the community,” “understanding the demands for community healthcare,” and “harmonization with community residents” were more effective in achieving the medium-term effects of CBME. The specific programs in the Tamba region included interviews with local staff, group discussions with hospital staff, exchange meetings with residents and staff, exchanges with residents at community centers, agricultural experiences under the guidance of residents, interviews with residents, and homestay practices at residents’ homes. Particularly, in the Tamba region, homestay training was conducted in the homes of residents, which has not been implemented in other regions [11,12,20]. In homestay practice, host families were determined via open recruitment, and students and residents lived together intensively for two days and one night, providing an opportunity to be in contact with each other for a long time [20]. Educational programs that enhance interactions with the population can potentially strengthen the relationship between local doctors and their patients. However, no data support the effectiveness of such medical programs worldwide [21]. In this study, we demonstrated a medium-term educational effect of CBME for a short period of three days and two nights only in the Tamba region. This was largely because the education program reinforced interactions with residents. This study demonstrates the usefulness of an educational program that strengthens interactions with the local population. There was no outreach activity or faculty involvement in the creation of CBME in each training site. Therefore, it is unlikely that differences in initiatives other than CBME influenced the students' decision-making.

This study has some limitations. First, the study population was limited to regional quota students. Additionally, the number of hospitals where regional quota students can apply for initial training is limited to 10 hospitals within Hyogo Prefecture, and the four hospitals presented here are all part of this group. General medical students who are not regional quota students are free to choose any of the more than 500 initial training hospitals in Japan; therefore, the results of this study are not necessarily applicable to general medical students.

The choice of hospital for the residency program is based on students’ preferences; however, the final decision is made by the relevant department in Hyogo Prefecture. This indicates that factors beyond students’ personal preferences exist. Therefore, no significant differences were found in the actual initial training locations.

This study compared the effectiveness of Tamba’s program to other programs in terms of practical training content. However, this comparison of student application results and CBME practice content may have other confounding factors. “Other unmeasured factors (e.g., brand strength of Tamba Hospital, attractiveness of senior doctors, location, etc.)” may have influenced the motivation of students to apply. We did not directly ask students in detail why they applied to become resident doctors in each hospital. Therefore, the next step is to directly ask students whether their satisfaction with CBME plays a role in their decisions to apply to become a resident doctor. As this is an observational study, causality cannot be ruled out.

The factors “affect heuristic” and “framing effect,” which are part of the category “psychological effects” [15], motivating medical students toward community medicine, were not examined in this study. The “affect heuristic” and “framing effect” were excluded because they are based on personal feelings and are difficult to classify based on the training content in this study. These are the contents that will be necessary in the next step when we directly ask students about their motivations for choosing their career paths.

Future research on CBME methods will need to go beyond simply visiting the rural community and will need to incorporate intensive interaction with local residents. In particular, it is believed that mid-term effects of the internship will be achieved by students having “empathy for the region,” “understanding of universal demand for community healthcare,” and “practice time with the resident community,” and spending more time in the internship interacting with the local community. Specifically, the training content being considered will include interviews with staff from the local area, as shown in Appendix A; group discussions including hospital staff; social gatherings with residents and staff; interactions with residents at community centers; agricultural experiences under the guidance of local residents; interviews with residents; and homestays in residents' homes.

## Conclusions

The medium-term educational effect of CBME on the choice of residency training location was observed only at the Hyogo Prefectural Tamba Medical Center, where participants who experienced CBME in their fourth to sixth year of medical studies expressed a preference for being matched for initial training. This proved the effectiveness of the CBME program in Tamba (education and training) for becoming a resident doctor in this region. Additionally, by comparing with other regions, the characteristics of CBME content in Tamba were identified. The factors “empathy for the region,” “understanding of universal demand for community healthcare,” and “practice time with the resident community” were significantly longer in the hospital of the Tamba region than in the other three regional hospitals considered in this study. This may help explain why CBME produced medium-term educational effects even during the short CBME period in the Tamba region. The CBME, which incorporates practical training content to enhance interaction with local residents, is an educational program designed to strengthen community engagement. To the best of our knowledge, this is the first study to demonstrate the effectiveness of such a program. However, this is an observational study, and factors other than the increased desire for community medical care may be involved. As a next step, further research, such as the direct questioning of participants, is needed.

## Appendices

Appendix A 

**Table 9:** Tamba region practical training/hands-on experience: 2019 Practical training applicable to (1), (2), and (7) is in bold.

Date	Time	Place	Activity contents	Correspondence with content (1)-(7)
August 22 (Thursday)	10:00 am –	Kobe University	About the system of regional quota students – doctors in Hyogo	
	1:00 pm–	Kobe University	Kobe University School of Medicine Center for Advancement of Community Medicine	
	1:00–2:30 pm	Move to Tamba		
	2:30–2:45 pm	Tamba Medical Center (TMC)	Orientation by Dr. K, Head of the Center for Community-Based Medical Education	
	2:50–4:45 pm		Medical experience training: Divided into 3 groups. Group 1: Ultrasound practical, Group 2: Cardiopulmonary resuscitation (CPR), Group 3: Mock interviews across 3 programs in rotation.	(3)
	4:45–5:00 pm		Round table conference with senior residents belonging to the doctor education system in Hyogo	(3)(4)(5)
	5:00–5:30 pm		Lecture by the mayor of Tamba city (charm and appeal of Tamba, and medical care issues, etc.)	(1)(2)
	5:30–5:40 pm		Break (set up the gathering)	
	5:40–6:45 pm		Gathering with senior residents, doctors, staff, etc.	(1)(2)(7)
	6:45–7:30 pm	Move to	Homestay location	
	7:30 pm –	Accommodation homestay location	Dinner with host family, hearing survey on health, Relaxing time (take a bath, help yourself)	(1)(2)(7)

Date	Time	Place	Activity contents	Correspondence with content (1)-(7)
August 23 (Friday)	8:20 am –	Accommodation	Breakfast at the homestay location	
	8:20–9:00 am	Move to TMC	From Homestay location to Tamba Medical Center	
	9:00–9:30 pm	Tamba Medical Center (TMC)	Lecture by the president of Tamba Medical Center (overall about the hospital, etc)	(3)
	9:30–10:15 am		Interview with the local staff who is from Tamba	(1)(2)(3)(7)
	10:15–10:30 am		Round table conference with fellows belonging to the doctor education system in Hyogo	(3)(4)(5)
	10:40 am–12:30 pm		Group discussion and presentation on medical issues and solutions in Tamba *TMC executives and others attend the meeting	(2)(3)(6)(7)
	12:30–1:15 pm		Lunch at TMC cafeteria/break time	
	1:15–1:40 pm	(Move to)	From TMC to Kagura-no-Sato	
	1:40–3:40 pm	Kagura-no-Sato	Students participated in local community activities: join the Exercise “Iki-Iki Hyakusai Taiso”, take blood pressure, hearing survey on health, tea talk party with local inhabitants etc, lecture to the local inhabitants	(1)(2)(3)(7)
	3:40–3:50 pm	(move to)	from Kagura-no-Sato to Aogaki region	
	3:50–5:40 pm	Hotaru-no-Sato Aogaki Region	Activities by local instructors: Agricultural experience, planting vegetables, catching fish “Amago” red-spotted trout	(1)(6)(7)
	5:40–6:30 pm	(Move to)	from Aogaki to Inaagki-cho (break and help to set up the gathering)	
	6:30–8:30 pm	Iso Community Center	Gathering with TMC doctors, local community clinic doctors, inhabitants, and so on. Event: “Nagashi Somen” Japanese thin wheat noodle flowing down a bamboo chute by Tamba Medical care reproduction network	(1)(2)(6)(7)
	8:30–8:40 pm	(Move to)	From the Iso Community Center to Accommodation	
	8:40 pm –	Accommodation	Yamada Ryokan (Hikami-cho, Tamba city) Interaction and exchange of opinions between students in the regional quota	(6)

Date	Time	Place	Activitycontents	Correspondence with content (1)-(7)
August 24 (Saturday)	8:30 am –	Accommodation Yamada Ryokan	Breakfast at Yamada Ryokan	
	8:30–8:45 am	(Move to)	From accommodation to Kasuga-cho	
	8:45–11:00 am	Kasuga-cho, Tamba city	Stroll around and explore Kasuga-cho, Tamaba Temple “Kozenji”, Castle “Kuroi jyo” Ruins (visit to places of Historical drama by Japan Broadcasting Corporation in 2020, the main character “Akechi Mitsuhide” 明智光秀)	(1)(6)
	11:00 am–12:15 pm	(Move to)	From Tamba city to Kobe	
	12:15 pm–	Kobe University	Kobe University School of Medicine Center for Advancement of Community Medicine (Lunch break at Kobe University CACM) 1:10 pm: Orientation, ready for presentation 3:30–6:00 pm Presentation at Auditorium 2F 6:30–8:00 pm Information exchange session	
